# Digital CBTI hubs as a treatment augmentation strategy in military clinics: study protocol for a pragmatic randomized clinical trial

**DOI:** 10.1186/s13063-023-07686-2

**Published:** 2023-10-06

**Authors:** Anne Germain, Megan Wolfson, Matthew S. Brock, Brian O’Reilly, Hunter Hearn, Shelley Knowles, Vincent Mysliwiec, Meredith L. Wallace

**Affiliations:** 1 NOCTEM, LLC, 218 Oakland Avenue, Pittsburgh, PA 15213 USA; 2 Department of Sleep Medicine, Wilford Hall Ambulatory Surgical Center, 1100 Wilford Hall Loop, Lackland AFB, San Antonio, TX 78236 USA; 3 Madigan Army Medical Center, Joint Base Lewis-McChord, WA, 9040 Jackson Ave., Joint Base Lewis-McChord, WA 98431 USA; 4 Carl R. Darnall Army Medical Center, Sleep Disorder Center, 36065 Santa Fe Ave., Fort Hood, Fort Cavazos, TX 76544 USA; 5 Department of Psychiatry and Behavioral Sciences, University of Texas Health Science Center at San Antonio, San Antonio, TX 78229 USA; 6 Department of Psychiatry, University of Pittsburgh School of Medicine, Pittsburgh, USA

**Keywords:** Chronic insomnia, Cognitive behavioral therapy for insomnia (CBTI), Digital technology, Digital CTBI hubs, Military treatment facility, Military, Veteran

## Abstract

**Background:**

Chronic insomnia is the most prevalent sleep disorder among military service members, and it compromises readiness, performance, and physical and mental health. Cognitive behavioral treatment for insomnia (CBTI) is the standard of care for the treatment of insomnia recommended by the VA/DoD Clinical Practice Guideline, the American Academy of Sleep Medicine, and the American College of Physicians. CBTI is highly effective but has limited scalability. It is often unavailable in clinical settings where service members receive sleep care. Digital technologies offer unique opportunities to scale and broaden the geographic reach of CBTI services and support increased patient access and engagement in behavioral sleep care. This study aims to evaluate the impact and acceptability of digital CBTI hubs to augment military treatment facilities’ capabilities in behavioral sleep medicine.

**Methods:**

This is a multi-site, non-inferiority randomized clinical trial designed to compare the effects of in-person (face-to-face or virtual) insomnia care as usual at three military sleep clinics versus CBTI delivered remotely and asynchronously through digital CBTI hubs. Digital CBTI hubs are led by licensed, certified clinicians who use NOCTEM’s® evidence-based clinical decision support platform COAST™ (Clinician Operated Assistive Sleep Technology). Changes in insomnia severity and daytime symptoms of depression and anxiety will be compared at baseline, at 6–8 weeks, and at 3-month follow-up. Patient satisfaction with insomnia care as usual versus digital CBTI hubs will also be examined. We hypothesize that digital CBTI hubs will be non-inferior to insomnia care as usual for improvements in insomnia and daytime symptoms as well as patient satisfaction with insomnia care.

**Discussion:**

Digital technology has a high potential to scale CBTI accessibility and delivery options required to meet the insomnia care needs of military service members. Digital CBTI hubs using COAST offer a novel approach to broaden service members’ access to CBTI and to serve as an augmentation strategy for existing sleep services at military treatment facilities. The pragmatic approach leveraging technology in this trial has the potential to rapidly inform clinical practice within the Defense Health Agency as well as other healthcare systems.

**Trial registration:**

ClinicalTrials.gov NCT05490550. Registered on 14 July 2023.

## Administrative information

Note: The numbers in curly brackets in this protocol refer to the SPIRIT checklist item numbers. The order of the items has been modified to group similar items (see http://www.equator-network.org/reporting-guidelines/spirit-2013-statement-defining-standard-protocol-items-for-clinical-trials/).

**Table:** 

Title {1}	Digital CBTI hubs as a treatment augmentation strategy in military clinics: study protocol for a pragmatic randomized clinical trial
Trial registration {2a and 2b}	ClinicalTrials.gov: NCT05490550
Protocol version {3}	Date: 08 June 2023 Version: v1.6; Original
Funding {4}	This trial is funded by the Joint Warfighter Medical Research Program (JWMRP).
Author details {5a}	**Corresponding Author**: *Anne Germain, Ph.D.* NOCTEM, LLC, 218 Oakland Avenue, Pittsburgh, PA 15213 anne@noctemhealth.com *Megan Wolfson, LCSW* NOCTEM, LLC, 218 Oakland Avenue, Pittsburgh, PA 15213 megan@noctemhealth.com *Matthew S. Brock, MD* Department of Sleep Medicine, Wilford Hall Ambulatory Surgical Center, 1100 Wilford Hall Loop, Lackland AFB, TX 78236 matthew.s.brock.mil@health.mil *Brian O’Reilly, DO* Madigan Army Medical Center, Joint Base Lewis-McChord, WA. 9040 Jackson Ave, Joint Base Lewis-McChord, WA 98431 brian.oreilly3.civ@health.mil *Hunter Hearn, MD* Carl R. Darnall Army Medical Center, Sleep Disorder Center, 36065 Santa Fe Ave, Fort Hood, TX 76544 hunter.a.hearn.civ@health.mil *Shelley Knowles, MD* Carl R. Darnall Army Medical Center, Sleep Disorder Center, 36065 Santa Fe Ave, Fort Hood, TX 76544 shelley.r.knowles.civ@health.mil *Vincent Mysliwiec, MD* Department of Psychiatry and Behavioral Sciences, University of Texas Health Science Center at San Antonio, San Antonio, TX 78229 vmysmd@gmail.com *Meredith L. Wallace, Ph.D.* University of Pittsburgh School of Medicine, Department of Psychiatry merewallace@gmail.com
Name and contact information for the trial sponsor {5b}	NOCTEM, LLC Contact name: Dr. Anne Germain Email: anne@noctemhealth.com Telephone: (412) 897-3183
Role of sponsor {5c}	Employees of NOCTEM, LLC conceptualized the study design and/or statistical plan. NOCTEM, LLC will have no interaction with the study participants, and statistical analysis will be conducted by an independent biostatistician. All publications will be reviewed by site investigators prior to submission.

## Introduction

### Background and rationale {6a}

Chronic insomnia is pervasive among service members and degrades health and military readiness. Chronic insomnia is defined as difficulty falling or staying asleep that persists for at least 3 months and that results in one or more impairments in daytime functioning (e.g., memory and concentration difficulties; irritability; somatic symptoms; worry; absenteeism) [1]. Insomnia is the most common sleep disorder in military personnel, affecting approximately 20% of service members, with reported rates as high as 63% [2–5]. Marked increases in insomnia have been observed over the past two decades in service members in all branches of the US military, with military health surveillance data showing a 50-fold increase in the diagnosis of insomnia between 2005 and 2019 [6]. The consequences of insomnia on military effectiveness are well documented and result in increased healthcare utilization, lower rates of deployment, and higher rates of mental health disorders [7, 8]. Insomnia often occurs comorbidly with psychiatric and medical conditions including anxiety, depression, PTSD, TBI, suicidality, alcohol use disorder, hypertension, and diabetes and compromises performance, readiness, resilience, and safety [9–14]. Insomnia rarely remits on its own, and treatment is required for symptom improvement and remission [15, 16].

Cognitive behavioral treatment of insomnia (CBTI) is the first-line insomnia treatment. The VA/DoD, the American College of Physicians, and the American Academy of Sleep Medicine have all concluded that CBTI is the treatment of choice for chronic insomnia [1, 17, 18]. Non-pharmacological, behaviorally based treatments for insomnia are also generally preferred by patients [19–23]. For example, in a qualitative study of veterans with insomnia, 80% reported having negative prior experiences with sleep medications. In this same study, most veterans reported being receptive to behavioral insomnia treatment approaches, and all felt they could reasonably partake in CBTI sessions [23]. Despite the effectiveness of CBTI and patient preference for this approach, CBTI is underutilized and often not available in settings where service members receive healthcare.

Digital health technologies can help to overcome the limited scalability of in-person healthcare services, including CBTI. There are two primary barriers to the access and delivery of CBTI that digital health technologies target. First, digital health technologies broaden the geographic reach of services. Providers with specialty training in CBTI are not available across the military treatment facilities and clinics in the USA and abroad where service members receive care. Even if a service member is located at a treatment facility with a sleep clinic, clinical resources are focused on sleep-disordered breathing and other sleep disorders (e.g., hypersomnolence, parasomnias), restricting the availability of CBTI offerings [24]. Despite efforts to increase the number of providers trained in CBTI, geographic limitations remain [25, 26]. Furthermore, the extent to which providers outside of a specialty sleep clinic who are trained in CBTI consistently add a CBTI component to structured group or individual outpatient treatment programs is also uncertain [27]. Thus, developing and leveraging digital health tools to support the provision and implementation of reliable sleep assessments and delivery of CBTI in a wider range of clinical settings and by a broader group of providers could help to support the insomnia treatment needs of service members.

Second, digital health technologies create opportunities for increased patient access and engagement with healthcare services. The majority of service members own a smartphone and thus have familiarity with the use of the tools required for digital healthcare services [28, 29]. Traditional CBTI treatment sessions typically range from 5 weeks to several months. Digital health technologies alleviate patient burdens of attending weekly or bi-weekly in-person (i.e., face-to-face or virtual) sessions and minimize if not eliminate the need to account for travel time to and from the clinic and coordination with work, family, or childcare responsibilities [30–34]. For some service members, it may also alleviate the stigma associated with engagement in behavioral health care [35, 36]. Several studies have highlighted that guided interactions with a clinician during the use of digital health tools are key for patient engagement and optimal clinical outcomes [37, 38]. However, most currently available digital CBTI offerings, including web-based self-guided programs and self-help apps, do not support real-time and/or just-in-time provider-patient interactions and supervision. As service members are a high-risk occupation, those receiving CBTI require easy access to a provider who is able to address not only their clinical needs, but also address their ability to safely perform their military duties. To meet the needs of service members with chronic insomnia, a strategy that maximizes the current advantages of digital CBTI technology (i.e., increased reach and decreased patient burden) and that offers high-quality, clinician-supervised, military focused, and streamlined services with minimal added demands for clinicians and their patients is needed.

Our proposed strategy is the extension of CBTI services through digital CBTI hubs to augment the military sleep clinic’s capabilities. A digital CBTI hub consists of a trained licensed clinician who will use the Clinician Operated Assistive Sleep Technology (COAST™) platform developed by the digital behavioral sleep medicine company NOCTEM to deliver asynchronous, remote, and personalized CBTI to service members referred by military sleep healthcare providers. Briefly, COAST is a cloud-based software platform that consists of (1) clinician portal with embedded algorithms for the detection of sleep-disordered patterns, clinical decision support tools, and evidence-based materials for the management of insomnia; (2) cross-platform patient mobile app that includes daily self-report sleep/wake logs, adherence measures, and weekly assessments of symptoms, side effects, and progress; and (3) secure, two-way HIPAA-compliant text messaging to support patient engagement, adherence, and supervision.

Digital CBTI hubs may offer a unique strategy for scaling delivery and access to CBTI in four distinct ways. First, digital CBTI hubs can serve as an extension of clinical services offered by sleep, behavioral health, or primary care clinics and maintain the continuity of care between the healthcare providers and patients. Direct referral of patients with insomnia to a digital CBTI hub creates an opportunity for personalization of the CBTI delivery format and allows clinicians at military clinics to offer face-to-face services to more complex patients. Second, digital CBTI hubs have a broad geographical reach and can overcome the current transportation and distance challenges for service members throughout the world. Third, they provide patients with just-in-time access to a CBTI expert for supervision, guidance, and clinical and military duty-related counseling when and as needed. Lastly, with COAST, they overcome many of the barriers posed by conventional in-person CBTI such as wasted time in session for computing sleep diary metrics via paper sleep diaries.

Accordingly, the goal of this paper is to describe the design and analysis plan of the clinical trial to evaluate and compare the effectiveness of digital CBTI hubs using COAST as an augmentation strategy for the management of insomnia at three military sleep clinics relative to insomnia care as usual (ICAU). This clinical trial is the first to evaluate the extent to which digital CBTI hubs can support patient outcomes and the acceptability of this referral approach among service members with insomnia.

### Objectives {7}

The overall objective of this trial is to evaluate the impact and acceptability of digital CBTI hubs to augment military treatment facilities’ capabilities in behavioral sleep medicine and maintain high-quality clinical outcomes. The specific aims are to (1) examine and compare the changes in insomnia severity at baseline, at 6–8 weeks, and at 3-month follow-up; (2) examine and compare the changes in daytime symptoms of depression and anxiety at baseline, at 6–8 weeks, and at 3-month follow-up; and (3) examine and compare patient satisfaction with ICAU versus CBTI via a digital CBTI hub.

The first hypothesis is that improvements in insomnia symptom severity in service members with a diagnosis of chronic insomnia following remote, asynchronous delivery of CBTI via a digital CBTI hub will be non-inferior to improvements detected following ICAU after 6–8 weeks and at 3-month follow-up. The second hypothesis is that improvements in daytime symptoms of depression and anxiety in service members with a diagnosis of chronic insomnia following remote, asynchronous delivery of CBTI via a digital CBTI hub will be non-inferior to improvements detected following ICAU after 6–8 weeks and at 3-month follow-up. The third hypothesis is that satisfaction with insomnia care following treatment through a digital CBTI hub will be non-inferior to ICAU.

### Trial design {8}

This is a multi-site phase II, parallel, non-inferiority randomized clinical trial that aims to compare the short- and medium-term effects of ICAU at three military sleep clinics versus CBTI delivered remotely through digital CBTI hubs. ICAU and the digital CBTI hubs will be evaluated and compared on an insomnia outcome measure as well as patient-rated treatment satisfaction and daytime mood and anxiety symptoms. In alignment with the pragmatic nature of this study and, if successful, the aim to rapidly inform insomnia care practices, this trial is designed to minimize disruption of each sites’ current processes for the diagnosis of insomnia and subsequent insomnia treatment procedures. All sites will follow their standard procedures for service members with complaints of sleep disturbances to obtain an appointment with the site’s sleep medicine clinic for an initial evaluation. Following this appointment, service members who receive a diagnosis of chronic insomnia by their sleep medicine provider and who would otherwise be referred (internally or externally) for CBTI will be referred to the study. After providing informed consent, eligible participants will complete baseline assessments including validated self-report questionnaires on insomnia, anxiety, depression, and sleepiness. Participants will then be randomized to either ICAU or insomnia care delivered via a digital CBTI hub. ICAU will be delivered synchronously, in-person (face-to-face or virtual) according to each site’s current standard of care and in the real-world clinical practice setting by a non-research clinician. While there are practice overlaps across sites, some variation exists in procedural and clinical treatment approaches and delivery formats (e.g., required attendance at a sleep seminar in addition to individual sessions, emphasis given to the cognitive component of CBTI, in-person versus synchronous telehealth, and scheduled session frequency). To maximize the generalizability of findings to current clinical practices, ICAU will not be standardized across sites or clinicians. All ICAU clinicians will deliver insomnia care to study participants as they would otherwise treat patients with chronic insomnia who are not participants in this clinical trial. Insomnia care delivered via the digital CBTI hubs includes the first-line recommended treatment for insomnia, CBTI, delivered remotely and asynchronously with NOCTEM’s clinical decision support platform called COAST by a NOCTEM-certified and licensed clinician. A follow-up will be conducted with participants randomized to both treatment arms 6–8 weeks after baseline and again 3 months later.

## Methods: participants, interventions, and outcomes

### Study setting {9}

The study will be conducted with service members across three military treatment facilities in the USA. The three military treatment facilities have a dedicated sleep clinic with at least one behavioral sleep medicine provider (physician and/or psychologist) and existing procedures and personnel for insomnia treatment.

### Eligibility criteria {10}

Potential participants will be identified by their sleep medicine provider during regularly scheduled appointments with the sleep clinic at their military treatment facility. The sleep medicine provider will only inform patients about the study who are active duty service members, at least 18 years old, have a current diagnosis of chronic insomnia, and are deemed appropriate for a course of CBTI. As service members often present with psychiatric and medical conditions that are commonly comorbid with chronic insomnia (e.g., depression, hypertension, obstructive sleep apnea), they will be eligible if these conditions are adequately treated or managed, including using medication(s) that has been unchanged for at least 1 month and will remain unchanged for the acute intervention phase. Service members using prescribed sleep medications will also be eligible to participate in the trial if they have been on the same medication(s) and at the same dose(s) for at least 1 month, and there are no expected changes over the course of the acute intervention phase. Medication usage and changes will be tracked prospectively and be considered in the analytical plan should changes occur.

Service members will be excluded from participation if they present with an untreated or under-treated comorbid sleep disorder that requires independent treatment or that may be exacerbated by CBTI interventions (e.g., obstructive sleep apnea, rapid eye movement (REM) sleep behavior disorder, or sleepwalking with a history of injury to self or others). Individuals will also be excluded if they are determined to be in acute psychiatric distress associated with marked impairments in functioning or that limits engagement in CBTI, adversely impacts the risk/benefit ratio of participating in the study, or requires immediate attention (e.g., attempted suicide in the past 6 months, seeking and/or receiving psychiatric treatment that requires inpatient or partial hospitalization level of care, requiring support of an assigned case manager for activities of daily living, active substance use disorder, past or current diagnosis or sub-threshold symptoms of psychotic or bipolar disorders, and untreated seizure disorder). Breastfeeding women or parents of an infant under the age of 3 months will also not be eligible, as their insomnia is confounded by these conditions, and CBTI may create greater-than-expected levels of transient sleepiness or fatigue. These minimal exclusion criteria are designed to support the recruitment of participants who are representative of adults with chronic insomnia and generalizable to real-life findings for military and civilian populations. The exclusion criteria that have been established are intended to minimize the risks associated with study participation. Nonetheless, each referring sleep medicine provider will use their clinical judgment to make determinations about the appropriateness of study participation, including CBTI for the patient, and will determine whether a patient is eligible for the study.

### Who will take informed consent? {26a}

After being informed of the study by their sleep medicine provider, service members who express interest in participating in the study will be informed of how to contact the site research coordinator directly or may agree to be contacted by the site research coordinator. At their first contact with the coordinator, the coordinator will review details about the study aims, procedures, and risks following a script to verify the consistency and completeness of the information provided. They will also answer any questions the potential participant may have about the study. If the person declines participation, the coordinator will contact the referring sleep medicine provider to let them know that the patient should be scheduled for follow-up care at the local sleep clinic. For participants who remain interested in participating in the study, after receiving information about the study and obtaining answers to questions that may have arisen, oral consent will be obtained to conduct a brief screening interview to confirm eligibility. Upon verification of eligibility, participants who express the desire to participate in the study will then review the consent form with the coordinator.

### Additional consent provisions for collection and use of participant data and biological specimens {26b}

As part of the informed consent, participants will confirm agreement to have their de-identified data maintained for future use, which may include further analysis and comparison of the dataset created in this study to others.

### Interventions

#### Explanation for the choice of comparators {6b}

As a non-inferiority trial, this study is designed to assess if digital CBTI hubs are no worse than the current standard therapy being offered to patients with chronic insomnia in military treatment facilities [39]. As such, we selected to compare the digital CBTI hubs to the current insomnia treatment practices (insomnia care as usual (ICAU)) at participating military treatment facilities.

#### Intervention description {11a}

Both intervention delivery formats, ICAU, and digital CBTI hubs using COAST will be delivered by credentialed masters or doctoral-level clinicians with experience in behavioral sleep medicine and include CBTI techniques. CBTI is a multi-component manualized cognitive-behavioral treatment protocol for the treatment of insomnia that is widely available. Among the various techniques that can be combined into CBTI, the most effective techniques to reduce insomnia are stimulus control and sleep restriction [40, 41]. Stimulus control aims to limit the use of the sleep environment (bed, bedroom) to sleep and directly addresses behaviors that disrupt circadian sleep–wake regulation mechanisms [42–44]. Sleep restriction involves the implementation of a regular sleep–wake schedule, which limits the time spent in bed while awake [45]. Relaxation techniques (e.g., progressive muscle relaxation, deep breathing, guided imagery) may also be helpful to reduce cognitive or physical hyperarousal that delays sleep onset [17].

Specifically, ICAU will include the usual care for service members diagnosed with insomnia at their local military treatment facility, as per the site’s current clinical practices. Treatment sessions will be conducted with the treating clinician(s) who typically receive insomnia referrals. Across sites, treatment duration will range from approximately 6 to 12 weeks with appointments scheduled every 1 to 3 weeks. Sleep metrics and progress will be evaluated and assessed by sleep diary, validated measures such as the Insomnia Severity Index and Epworth Sleepiness Scale, and patient self-report during scheduled sessions. All sessions will be conducted synchronously and in person (face-to-face, virtually). Patients may be required to attend a single sleep hygiene group seminar where foundational healthy sleep habits and relaxation skills are reviewed.

Treatment via a digital CBTI hub includes CBTI delivered remotely and asynchronously via NOCTEM’s proprietary software platform COAST (Clinician Operated Assistive Sleep Technology) with a NOCTEM research clinician. All research clinicians are fully credentialed, licensed healthcare professionals certified in the use of COAST by NOCTEM. COAST is an adaptive digital health system that includes a clinician portal for prospective monitoring of side effects, progress, and adherence and algorithm-generated recommendations for the implementation of CBTI techniques; a patient application (“app”) with embedded sleep educational materials and self-report daily sleep diary and weekly assessments; and a secure HIPAA-compliant two-way messaging system that allows patients and clinicians to communicate directly in case of questions. Treatment sessions will be conducted across 6–8 weeks, with asynchronous clinical interactions occurring throughout the 6–8-week period with at least one interaction per week. Minimally, each week, the research clinician will send personalized recommendations for behavioral changes to the participant via the app and instruct the participant to review specific sleep tactics (written or audio CBTI interventional materials) that provide instruction on how to implement and the background for the CBTI techniques. Dialog regarding these recommendations will primarily be conducted via the COAST messaging system; however, at any time during the intervention, the participant or the clinician may request a synchronous telephone or in-person appointment.

#### Criteria for discontinuing or modifying allocated interventions {11b}

All clinicians will use their clinical judgment to assess the ongoing appropriateness of the participant for CBTI. Should it be determined that the participant is no longer a good candidate for CBTI, the participant’s treatment will be discontinued, and study participation terminated.

#### Strategies to improve adherence to interventions {11c}

This is a pragmatic trial. While site coordinators will facilitate the initial steps for receipt of care for ICAU and the digital CBTI hub, no study-specific standardized strategies or procedures will be used to ensure participant attendance or compliance with treatment sessions or CBTI techniques. ICAU clinicians will continue any existing strategies typically used at their site and that they have found helpful to support adherence to treatment. Digital CBTI hub clinicians will use the just-in-time monitoring capabilities embedded in the COAST platform and prompt participants when they have missed logging their sleep on the COAST patient app. The COAST patient app includes reminders to complete the sleep diary to facilitate data collection.

#### Relevant concomitant care permitted or prohibited during the trial {11d}

Participants may receive care for comorbid psychiatric and medical conditions to the extent that eligibility criteria for the trial continue to be met. Specific details are provided above in the “Eligibility criteria {10}” section.

#### Provisions for post-trial care {30}

The anticipated risks for participants are believed to be minimal. Throughout this study, all procedures and techniques involve no more than minimal risk and are the same as would be expected from the traditional in-person sleep intervention. The participant’s sleep provider will make determinations about any further care required for the participant following a course of CBTI or if it is deemed participation in CBTI should be terminated.

### Outcomes {12}

The primary outcome measure is the Insomnia Severity Index (ISI) [46, 47]. The ISI is a 7-item self-report questionnaire that assesses the subjective severity of insomnia symptoms, degree of satisfaction with sleep, and nature and noticeability of daytime impairments. Each item is rated on a 0 to 4-point scale. A score of 15 or greater reflects clinically significant insomnia. All participants will be administered the ISI at baseline, 6–8 weeks later, and then again at 3-month follow-up.

To assess the changes in depression and anxiety symptom severity, the Patient Health Questionnaire-2 items (PHQ-2) [48, 49] and the Generalized Anxiety Disorder-2 items Scale (GAD-2) [50] will be used. The PHQ-2 assesses depressed mood and anhedonia, which are the first two items of the 9-item version of the Patient Health Questionnaire. The score ranges from 0 to 6, and a total score of greater than 3 indicates likely major depressive disorder. The GAD-2 assesses the severity of feelings of nervousness and worry on a score of 0 to 3 for each of the 2 items. A total score of greater than 3 has been shown to have good sensitivity and specificity for anxiety disorders. All participants will be administered the PHQ-2 and the GAD-2 at baseline, 6–8 weeks later, and at 3-month follow-up.

Satisfaction with insomnia treatment will be assessed using two measures including an adapted version of the Insomnia Treatment Acceptability Scale (ITAS) [51] and the Client Satisfaction Questionnaire [52]. An adapted version of the ITAS will be used to assess overall perceived acceptability and treatment preferences for either of the two CBTI modalities. On the ITAS, both treatment options will be described, and participants will be asked to rate identically worded questions about the acceptability of the treatment for oneself and others, likelihood of adhering to treatment recommendations, and expectations of short- and long-term treatment effectiveness for specific insomnia symptoms (e.g., difficulty falling or staying asleep), expected impact of insomnia treatment on daytime symptoms of insomnia, and severity of expected side effects. These items are rated for both treatments, both of which are scored on a 10-point scale. The total score for each subscale is the average of the 8 items, with higher scores indicating greater willingness to utilize the treatment. Reported Cronbach’s values for the scales are greater than 0.80. For this trial, we will contrast responses to ICAU and digital CBTI hubs. The Client Satisfaction Questionnaire is an 8-item survey that assesses the perceived quality of care, services, and clinicians encountered during treatment. Higher scores indicate greater satisfaction. Both satisfaction surveys will be administered at 6–8 weeks only.

#### Participant timeline {13}

**Fig. 1:**
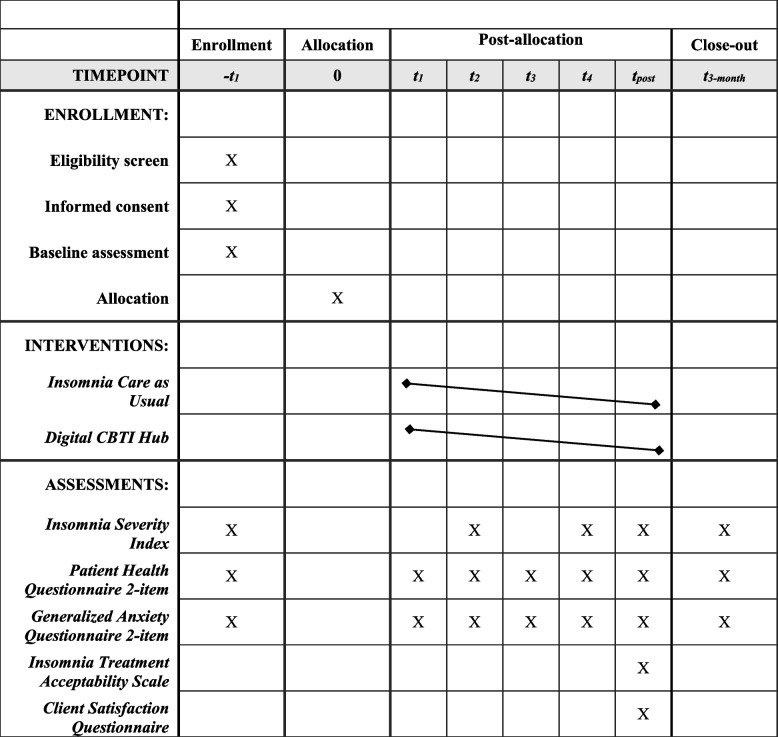
Schedule of enrollment, interventions, and assessments for study participants

The total duration of participation is up to 5 months. Figure 1 displays the schedule of enrollment, interventions, and assessments for the primary outcomes of interest.

#### Sample size {14}

Power calculations and sample size were based on a conservative estimate of the non-inferiority margin (NIM) based on our preliminary data and prior studies [53–55] and effect sizes reported in the literature on the effects of CBTI on insomnia severity [56–60], as well as estimates of recruitment feasibility based on patient volume at the participating sites. Specifically, effect sizes in the moderate to very large range (0.50 to 2.15) have been reported for insomnia, depression, and anxiety in military and civilian samples. Thus, we conservatively estimated a NIM of *d* = 0.5 for each outcome of interest for this trial. The NIM of |*d*|= 0.5 set to determine non-inferiority is stringent as it represents 50% of the effect size reported in recent meta-analyses [61–63]. For non-inferiority tests, we set an allowable one-sided type I error rate of *α* = 0.025. Conservatively adjusting for our two a priori *t*-tests (post-treatment and a 3-month follow-up), we use α* = 0.0125 to determine power. Based on the current patient volume and clinical workflows at the three participating sites, we estimated that a total of 188 patients could be enrolled in this clinical trial over the period of performance. Power calculations using PASS version 23.0.2. [64] yielded 88% power with a full sample of 94 participants per group (our primary strategy and 80% power with a final sample of 140 completers assuming an attrition rate of 25% (*N* = 70 completers per group).

#### Recruitment {15}

To facilitate recruitment in this trial, without interfering with the standard workflow of the military treatment facility providers and staff and patient care, each site will be supported by a research coordinator who will be responsible for consenting eligible participants and facilitating communication and processes between clinics and the digital CBTI hubs. We remain cautiously optimistic as the volume of patients diagnosed with insomnia annually at each of our participating sites represents more than 10 times our enrollment target.

### Assignment of interventions: allocation

#### Sequence generation {16a}

Eligible participants who provide informed consent will be stratified by the presence or absence of any current sleep, medical, or psychiatric comorbidities (yes/no) and the use of psychotropic medications (yes/no). Within each stratum, participants will be randomized in a 1:1 manner to ICAU or digital CBTI hub at the site level. Randomization within each stratum will follow a permuted block design (block size of three) to ensure that the treatments and clinicians are equally distributed across strata. The use of stratified randomization with permuted block randomization will provide a balance of treatment arms across psychiatric comorbidity and medication use. We considered stratifying on additional covariates (e.g., sex, age, enlisted vs. officer), but this would result in an unreasonably large number of strata, and there is little evidence that these may confound the outcome measures of interest. All analyses, however, will first consider plausible covariates. A computer-generated randomization scheme has been generated for each site.

#### Concealment mechanism {16b}

Access to the computer-generated randomization scheme will be limited to the site coordinator and only be accessed at the time of allocation to the treatment arm.

#### Implementation {16c}

Following participant consent to participate in the study, the coordinator will access the computer-generated randomization file to discover which treatment arm the participant has been randomized to. The participant and the referring sleep medicine provider will be informed of the randomization outcome. For participants randomized to ICAU, the coordinator will facilitate the scheduling of an appointment at the sleep clinic for ICAU through the referring site’s standard operating procedures. For participants randomized to the digital CBTI hub, the coordinator will facilitate a warm hand-off to the clinician affiliated with the digital CBTI hub, and the participant will then initiate CBTI under their close supervision.

### Assignment of interventions: blinding

#### Who will be blinded {17a}

Given the nature of the study design, blinding of participants or clinicians will not be possible. However, masked data will be provided to the biostatistician who will perform the analyses. Randomization will be unmasked only after analyses are finalized. Furthermore, clinicians providing ICAU or treatment via digital CBTI hubs will not participate in periodic interim analyses of the primary and secondary outcomes to avoid biases and/or changes in practice over the course of the study.

#### Procedure for unblinding if needed {17b}

Not applicable: neither enrolled patients nor clinicians will be blinded in this trial.

### Data collection and management

#### Plans for assessment and collection of outcomes {18a}

Participant data will be collected via HIPAA-compliant electronic data capture, including the NOCTEM COAST patient app. For all assessments on the app, validation checks are in place to ensure data quality and completeness. The research coordinators are trained on the use and functionality of the COAST patient app and will be available to participants should questions arise about using the technology to complete the assessments. Additional information about the study questionnaires can be found in the above section titled Outcomes.

#### Plans to promote participant retention and complete follow-up {18b}

The retention of participants for the follow-up period can be challenging. We will employ strategies that allowed us to complete post-intervention procedures and achieve high levels of adherence (> 80%) in previous studies, including, for example, participation gratitude letters [53–55].

#### Data management {19}

No hard copy data will be collected for this study. All participant information and assessments will be collected via HIPAA-compliant electronic data capture, including the COAST patient app. Security and confidentiality in a mobile health application are of paramount importance, and layered security measures are used to protect the privacy and confidentiality of the research data from the participants.

#### Confidentiality {27}

To protect confidentiality, each participant will have a research identification (ID) consisting of a 6-digit alpha-numeric code that will be used in lieu of personally identifiable information (name, date of birth, Social Security number, etc.) for data collection and storage. The research ID numbers are unrelated to any potentially identifiable numerical series, such as social security number, medical record number, or date of birth. Participant name and contact information will be collected for the purposes of contacting participants during study procedures. This information will be stored only for the duration of the study, stored separately from the study data, and deleted at the completion of the study. Study data will be kept strictly confidential, and participants’ identities will not be revealed in any publication. All data gathered in this project will be completed electronically, and data will be saved automatically into the main database. The AWS GovCloud managed by NOCTEM are cloud-based databases that are protected by several procedures, including password protection of participant data and a firewall.

#### Plans for collection, laboratory evaluation, and storage of biological specimens for genetic or molecular analysis in this trial/future use {33}

Not applicable: no biological specimens will be collected in this trial.

## Statistical methods

### Statistical methods for primary and secondary outcomes {20a}

Preliminary data analyses will be performed prior to testing the aims to check for collinearity of covariate and outcome measures, to ensure completeness and accuracy of data, and to evaluate distributions. The primary and secondary aims will be tested with non-inferiority hypotheses of the general form: H0: digital CBTI hubs are inferior to ICAU versus H1: digital CBTI hubs are non-inferior ICAU. For each outcome, we will calculate the standardized differences (Cohen’s *d*) as digital CBTI hubs—ICAU, such that a positive difference is in favor of ICAU if higher values are worse, and a negative difference is in favor of ICAU if higher values are better. Since higher values indicate worse outcomes for our measures of interest (e.g., ISI, depression, anxiety), we will use a non-inferiority margin (NIM) of 0.50 and declare digital CBTI hubs to be non-inferior to ICAU if the upper bound of the 95% confidence interval (CI) for *d* is ≥ 0.50. We will declare digital CBTI hubs to be inferior to ICAU if the upper bound of the 95% CI for *d* is ≤  − 0.50. For example, if *d* (95% CI) = 0.10 (− 0.20, 0.40), we would declare digital CBTI hubs as non-inferior to ICAU because 0.40 < 0.50. But if *d* (95% CI) = 0.40 (0.10, 0.70), we would declare digital CBTI hubs to be inferior because − 0.70 ≥ 0.50. These one-sided decision rules equate to requiring the effect size comparing digital CBTI hubs to ICAU to have no more than moderate strength (|*d*|= 0. 5) in favor of ICAU.

Our primary strategy will compare digital CBTI hubs and ICAU using analyses that are straightforward and highly interpretable while still accounting for other important covariates. We will use analysis of covariance (ANCOVA) to determine whether ISI at 6–8 weeks and at 3-month follow-up differs between digital CBTI hubs and ICAU after controlling for baseline ISI and other relevant covariates. Cohen’s *d* effect sizes (*d* ESs) with 95% CIs estimated from the ANCOVA model will be calculated and compared to the NIM as outlined above. To examine more nuanced effects across time, we will calculate the rate of change in ISI symptoms (ΔISI) across baseline, 6–8 weeks, and 3-month follow-up for each individual and then use linear regression to compare ΔISI between digital CBTI hubs and ICAU after controlling for relevant covariates. We will also model the repeatedly measured outcome using mixed effects models with a random intercept and slope. Cohen’s *d* ESs and 95% CIs estimated from these models will be calculated and compared to the NIM as outlined above.

For non-inferiority tests, we set an allowable one-sided type I error rate of *α* = 0.025. Conservatively adjusting for our two a priori *t*-tests (6–8 weeks and 3-month follow-up), we use α* = 0.0125 to determine power. Our full sample size of 94 per group will provide 0.88 power. However, even with attrition of 25% (*N* = 70 completers per group), we expect to have 0.80 power. We will use the same statistical approach to assess and compare changes in depression and anxiety symptom severity at 6–8 weeks and at 3-month follow-up.

### Interim analyses {21b}

Interim analyses will focus on enrollment and verification of data completeness and equivalence between the two intervention samples. No interim analyses are planned with the intent of potentially modifying study procedures or conduct.

### Methods for additional analyses (e.g., subgroup analyses) {20b}

If non-inferiority is established, we will conduct secondary tests to determine whether digital CBTI hubs may also be superior to ICAU.

### Methods in analysis to handle protocol non-adherence and any statistical methods to handle missing data {20c}

The primary analytic approach will be based on intent-to-treat principles and will include all individuals regardless of adherence to protocol, although “per-protocol” sensitivity analyses will also be conducted to evaluate the robustness of our findings. Missing observations will be investigated to determine the underlying mechanism (missing at random, completely at random, not at random) so that appropriate methods for handling the missing data can be employed.

### Plans to give access to the full protocol, participant-level data, and statistical code {31c}

The data sharing plan for this project will be based on requests from other researchers. Scientifically sound requests will be reviewed by the investigators, local IRB representatives, and the assigned Scientific Officer by the study sponsor.

We do not anticipate that unique data will be generated by this study. A final anonymized dataset will be generated from this study and will be made available to researchers. Requests to share the final research data will be evaluated individually for scientific merit, as described above. The sharing of data or resources for scientifically rigorous requests will follow the research practice standards and the sponsor’s policies. Each complete data sharing request will be carefully documented to ensure that the scope of work and intended use and publications are clearly outlined and agreed upon by all parties. A Data Use Agreement (DUA) will be completed by the requesting and sharing investigator and reviewed and approved by authorized representatives of both organizations. NOCTEM will maintain a record of these DUA and documentation of data sharing requests and approvals electronically. Completely de-identified and anonymized datasets will be shared through an encrypted FTP site. Documentation shared will include information about the study design, data collection methods, a code book defining fields and variables, and a dataset in Microsoft Excel file format. The shared data will be provided to the requester within 90 days of final approval, i.e., once the fully executed agreement signed by all parties involved is provided to the principal investigator.

### Oversight and monitoring

#### Composition of the coordinating center and trial steering committee {5d}

NOCTEM will be responsible for providing administrative, data management, and technical support to all three participating sites. Working closely with the three site coordinators, NOCTEM will hold weekly internal meetings to review study progress and communications received from the site investigators and site coordinators to promptly address any issue that may arise. With the oversight of site investigators, the site coordinators will be responsible for communicating and implementing any approved modifications to the study procedures and processes. While coordinators will be the primary points of contact for referral to the NOCTEM research clinician at the local digital CBTI hub, site investigators and research clinicians may communicate directly as needed to address participants’ needs or updates. NOCTEM will hold quarterly videoconference meetings with all COAST clinicians to review progress, address any questions that may arise, and communicate all relevant updates to the conduct of the study. Those members of the research team who are involved with the treatment of patients within the study will not review data that involve the comparison of outcomes (i.e., adverse effects, treatment response). The study is no greater than minimal risk, and therefore, an independent research medical monitor is not required.

While stakeholders and the public will not officially be involved in the operations of this trial, through, for example, a Stakeholder and Public Involvement Group (SPIG), efforts to engage and collect feedback from these entities will be ongoing. Opinions of military sleep medicine physicians were sought prior to finalizing the study procedures, and we will aim to keep appraised Defense Health Agency stakeholders and leadership of study developments, progress, and outcomes through channels outlined in our dissemination plan (see the “Dissemination plans {31a}” section). Comprehensibility of participant consent and information forms will be assessed at each patient encounter, and any issues or suggestions for improvements will be reviewed by the study team.

#### Composition of the data monitoring committee, its role, and reporting structure {21a}

Data and safety monitoring will be the shared responsibility of all members of the research team. The final responsibility will rest on the principal investigator. A data monitoring committee was not necessary for this trial because the procedures for sleep assessment, monitoring, and intervention being offered through the digital CBTI hubs reflect the digital version of tasks and procedures that would otherwise be completed during in-person visits for CBTI. The behavioral modifications being offered in ICAU and the digital CBTI hubs to promote consolidated and restorative sleep involve no more than minimal risk and are consistent with strategies recommended by the American Academy of Sleep Medicine and the VA/DoD Guidelines for the Management of Chronic Insomnia [1, 17].

#### Adverse event reporting and harms {22}

Should any adverse events occur in the study, the site investigator will be informed and report events immediately to the IRB. For service members, the anticipated risks are believed to be minimal. Participants will be updated if any new information is learned pertaining to unforeseen risks.

#### Frequency and plans for auditing trial conduct {23}

Review of the trial conduct will occur at regularly scheduled meetings with all investigators and research staff present and include assessment and discussion of study progress including data quality and timeliness and participant recruitment and retention, any factors impacting the anticipated risk/benefit ratio of study participation, and study privacy and confidentiality procedures.

#### Plans for communicating important protocol amendments to relevant parties (e.g., trial participants, ethical committees) {25}

Should it be determined that a protocol modification is required, the study team will act in accordance with current IRB or clinicaltrial.gov policies for reporting. If modifications include changes to the consent form, research coordinators will inform current participants of these revisions.

#### Dissemination plans {31a}

The findings of this pragmatic non-inferiority trial will be submitted for peer-reviewed publication. Primary and secondary data analyses will also be submitted for presentation at national scientific conferences. We also anticipate drafting a report on lessons learned and recommendations for the implementation of digital CBTI hubs to augment capabilities in behavioral sleep medicine which may be shared as part of discussion with stakeholders and leadership in the Defense Health Agency.

## Discussion

Significant disparities in access to the first line recommended treatment for insomnia, cognitive behavioral treatment for insomnia (CBTI), exist in the Defense Health Agency, including military treatment facilities with sleep clinics. The current lack of insomnia treatment for many service members contributes, at least in part, to worse physical and mental health outcomes while on active duty as well as when they become veterans. Several factors contribute to limited access to CBTI including high demands for services, a lack of trained providers, and rigid treatment structures. Digital health technologies create opportunities to scale access to CBTI, but existing self-guided programs and self-help apps shift the burden of care onto patients and disrupt the continuity of care which may not be appropriate for service members due to their high-risk occupation. Self-help apps and web-based self-paced programs also have low retention and treatment completion rates. COAST overcomes these barriers by providing just-in-time clinician-supervised CBTI. This pragmatic, non-inferiority trial is an important first step to evaluate the impact of digital CBTI hubs as a measure to extend the capability to offer CBTI at military clinics and ultimately non-military healthcare clinics.

Trial design selection is centered around maximizing the generalizability of results to clinical practice settings. In place of a superiority trial, we selected a comparative effectiveness, non-inferiority framework to enhance the potential to inform healthcare decisions by providing evidence of two treatment modality options—ICAU and digital CBTI hubs—versus the use of a wait-list control or inert condition. With a focus on ecological clinical validity, minimal exclusion criteria were selected, and those established were intended to minimize the risks associated with study participation. It was also determined that ICAU would not be standardized across sites or across clinicians to capture existing variability in CBTI offerings. Thus, clinicians at each military treatment facility will deliver insomnia care to study participants as they would otherwise as part of their typical clinical duties and site standards of care. If successful, with our pragmatic approach, the anticipated knowledge and service model tested in this trial has the potential to rapidly inform, support, and extend clinical practice and has a direct impact on clinicians and service members affected by insomnia throughout the Defense Health Agency and beyond.

While this trial is taking place only in military treatment facilities with sleep clinics, digital CBTI hubs could also be an appropriate augmentation strategy to support sleep healthcare services across the Defense Health Agency and, more broadly, for the Veterans Health Administration and civilian healthcare providers and organizations. While in-person CBTI is required for patients who present with severe insomnia and complex comorbidities, a significant portion of adults with insomnia show clinically meaningful improvements in sleep quality when adopting clinician-assisted digital sleep solutions. An arsenal of options for delivering evidence-based insomnia treatment recommendations (e.g., in-person, virtual, asynchronous clinician-assisted program, self-management apps) is required to meet the insomnia care needs of service members, as well as of veterans and the public. A broader range of treatment delivery options that optimize the capabilities of available resources and assets and that are sensitive to patients’ individual needs, resources, and constraints (e.g., travel, duty, appointment frequency, duration, location) is key to cost-efficient, wide-reaching strategies to prevent, rapidly detect, and treat insomnia.

## Trial status

This study received ethics review and approval from Madigan Army Medical Center’s Institutional Review Board (Protocol #223088) on 8 June 2023 and from the Human Research Protection Office (HRPO) (JW210372). Recruitment is anticipated to begin in August 2023 and be completed by April 2024.

## Data Availability

All data will be encrypted and stored in the AWS GovCloud owned by NOCTEM. A Collaborative Research and Development Agreement (CRADA) is in place with site investigators so that site-specific data collected can be shared.

## Abbreviations

ANCOVA: Analysis of covariance
CBTI: Cognitive behavioral therapy for insomnia
COAST: Clinician Operated Assistive Sleep Technology
*d* ES: Cohen’s *d* effect size
CI: Confidence interval
DUA: Data Use Agreement
GAD-2: Generalized Anxiety Disorder 2-item Scale
HIPAA: Health Insurance Portability and Accountability Act
HRPO: Human Research Protection Office
ICAU: Insomnia care as usual
ISI: Insomnia Severity Index
ITAS: Insomnia Treatment Acceptability Scale
NIM: Non-inferiority margin
PHQ-2: Patient Health Questionnaire 2-items
SPIG: Stakeholder and Public Involvement Group
